# Proposing diagnostic reference levels for paediatric computed tomography in Jordan: a national multicentre analysis

**DOI:** 10.1007/s13246-026-01731-5

**Published:** 2026-03-30

**Authors:** Abdel-Baset Bani Yaseen, Jamie Trapp, Davide Fontanarosa

**Affiliations:** 1 School of Clinical Sciences, Faculty of Health, Queensland University of Technology, Kelvin Grove Campus, Brisbane, QLD 4059 Australia; 2 Centre for Biomedical Technologies, Queensland University of Technology, Brisbane, QLD 4000 Australia; 3 School of Chemistry and Physics, Faculty of Science, Queensland University of Technology, Gardens Point Campus, Brisbane, QLD 4000 Australia; 4 Department of Medical Imaging, Faculty of Applied Medical Sciences, The Hashemite University, Zarqa, 13133 Jordan

**Keywords:** Diagnostic reference levels, Paediatric computed tomography, Radiation dose, Dose optimisation, Radiation protection

## Abstract

Children are more radiosensitive than adults, making dose optimisation in paediatric computed tomography (CT) essential. Although Diagnostic Reference Levels (DRLs) are internationally recommended as optimisation tools, national DRLs for paediatric CT in Jordan remain limited and outdated. This study aimed to establish national DRLs for six paediatric CT protocols, evaluate inter-hospital and age-related dose variations, and compare results with international benchmarks. A retrospective multicentre study was conducted across six Jordanian hospitals between December 2023 and September 2025, including 3794 paediatric patients stratified into four age groups (< 1, 1–4, 5–10, and 11–18 years). Volumetric CT dose index (CTDI_vol_) and dose-length product (DLP) data were collected for six protocols: brain, chest, abdomen–pelvis, chest–abdomen–pelvis, sinuses, and contrast-enhanced neck soft tissue. DRLs were defined as the 75th percentile of institutional median CTDI_vol_ and DLP values. Inter-hospital and age group variations were analysed, and univariable regression analyses assessed acquisition parameters associated with dose variation. Dose increased with patient age for trunk protocols, whereas brain, sinuses, and neck CT showed comparatively stable patterns. Substantial inter-hospital variability was observed across protocols, with institutional median CTDIvol differing markedly between centres, particularly for trunk examinations in younger age groups. In univariable regression analyses, all four acquisition parameters (kVp, mAs, pitch, and slice thickness) were significantly associated with CTDI_vol_ and DLP (*p* ≤ 0.001), with kVp demonstrating the strongest association (R^2^ = 0.603 for CTDI_vol_; R^2^ = 0.630 for DLP). Compared with published international DRLs, Jordanian brain CT dose metrics were higher in multiple age groups; chest comparisons should be interpreted cautiously where international benchmarks are weight-banded. This study established national paediatric CT DRLs for Jordan, highlighting the need for standardised protocols and periodic DRL review to enhance radiation protection.

## Introduction

Computed tomography (CT) is widely used in clinical imaging due to its high spatial resolution and diagnostic efficacy. However, its contribution to collective radiation dose is substantial, particularly in paediatric populations, who are more radiosensitive than adults due to their developing tissues and longer life expectancy [1]. As a result, children are more vulnerable to radiation-induced effects, underscoring the importance of dose optimisation in paediatric CT imaging [2, 3].

Globally, the sharp increase in CT utilisation has been recognised as a major source of medical radiation exposure. According to the United Nations Scientific Committee on the Effects of Atomic Radiation, CT accounts for approximately 9.6% of all medical radiological examinations, yet contributes about 61.6% of the overall collective effective dose from medical radiological exposure [4].

Diagnostic Reference Levels (DRLs) are widely endorsed as an optimisation tool in radiological protection. While not regulatory limits, DRLs are typically set at the 75th percentile of dose distributions obtained from routine clinical practice. Their purpose is to identify unusually high doses and trigger local review of imaging protocols [5]. The International Commission on Radiological Protection (ICRP) recommends that DRLs be reviewed and updated at regular intervals, generally every three to five years, to reflect changes in technology, clinical practice, and patient demographics [5]. When implemented effectively, DRLs can support dose reduction through standardisation and optimisation of protocols. In the United Kingdom, a national paediatric CT dose audit proposed updated DRLs for head CT that were lower than previous UK NDRLs by approximately 18–41% (depending on age band), whereas the same audit reported insufficient data to propose national DRLs for abdomen–pelvis CT [6].

In Jordan, national data on paediatric CT radiation exposure remain scarce. A previous study by Rawashdeh et al*.* (2019) proposed local DRLs for four CT examination types: brain, chest, abdomen–pelvis, and chest–abdomen–pelvis across four hospitals. Their findings showed considerable variability in the volumetric CT dose index (CTDI_vol_) and Dose Length Product (DLP) values between centres, with up to a four-fold difference for some procedures. In general, DLP values in Jordan were relatively high compared to international benchmarks, indicating the need for more consistent and optimised scanning protocols [7]. More recently, Hamedat et al*.* (2025) conducted a multicentre national survey of paediatric head CT across eight Jordanian hospitals and proposed age-based national DRLs, reporting substantial inter-hospital variability and underscoring the need for dose standardisation [8].

The present study builds upon and substantially extends the earlier work, providing more recent data with broader national representation. While the previous study was limited to four hospitals and four CT examinations, the current study includes six major hospitals and six paediatric CT procedures, representing a markedly larger and more comprehensive dataset. This expansion enables a more robust assessment of variations in CTDI_vol_ and DLP, while the proposed DRLs are benchmarked against internationally reported values to support optimisation and harmonisation of paediatric CT protocols in Jordan.

This study therefore aims to establish updated national DRLs for paediatric CT in Jordan, assess inter-hospital and age-related variations in CTDI_vol_ and DLP, and compare the proposed DRLs with international benchmarks to support dose optimisation and protocol harmonisation.

## Methods

### Study design

This multicentre, retrospective study was conducted to propose updated national DRLs for paediatric CT examinations in Jordan. Data were collected from December 2023 to September 2025 across six hospitals strategically selected to represent different geographical regions: two from northern Jordan, two from the central region, and two from the south. The selection of hospitals was based on their geographic distribution, technological diversity, and commitment to quality assurance programmes. A purposive sampling strategy was adopted to ensure representativeness across Jordan’s healthcare regions (north, central, and south). In addition, hospitals were included based on the availability of sufficient paediatric CT data, as paediatric examinations were not routinely performed in all centres. This approach ensured that the participating hospitals reflected both regional diversity and practical feasibility for comprehensive data collection.

In accordance with the conditions of ethical approval obtained from the Jordanian Scientific Research Ethics Committee, the names of participating hospitals have been anonymised and are referred to as Hospitals A to F throughout the manuscript.

The CT equipment varied in manufacturer and model, reflecting the technological diversity of participating institutions. The distribution was as follows: two Philips Brilliance 64-slice scanners (Philips Healthcare, Best, The Netherlands) at Hospitals A and B; one Siemens Somatom Definition 128-slice scanner (Siemens Healthineers, Erlangen, Germany) at Hospital C; one GE Revolution EVO 64-slice scanner (GE Healthcare, Chicago, IL, USA) at Hospital D; one Toshiba Aquilion Dual 64-slice scanner (Canon Medical Systems, Otawara, Japan) at Hospital E; and one Philips Brilliance 128-slice scanner (Philips Healthcare, Best, The Netherlands) at Hospital F.

At each site, a clinical coordinator with expertise in CT imaging was assigned to facilitate data collection. Standardised and pre-validated data collection forms were used across all hospitals to ensure consistency and reliability.

The study focused on six commonly performed CT examinations in paediatric imaging: brain, chest, abdomen–pelvis, chest–abdomen–pelvis, sinuses, and neck soft tissue. All examinations were acquired without intravenous contrast, except for neck soft tissue, which was performed with contrast. Examination selection was based on frequency of use and clinical relevance across the participating hospitals.

### Dose data and analysis

Radiation dose information was extracted directly from the Picture Archiving and Communication Systems (PACS) and Radiology Information Systems (RIS) of all participating hospitals using a standardised data-collection template. Extracted variables included patient age (years), sex, CT protocol, and scanner acquisition parameters (kilovoltage peak (kVp), tube current time product (mAs), pitch, field of view (FOV), and slice thickness), together with the corresponding dose indices (CTDI_vol_ and DLP). All data were anonymised prior to analysis. Patients were stratified into four age categories (< 1 year, 1–4 years, 5–10 years, and 11–18 years). International paediatric DRL guidance recommends stratifying patients using discrete age bands and/or body-size surrogates (commonly weight bands for trunk examinations). European guidance commonly applies age points up to 15 years (0, 1, 5, 10 and 15 years) and recommends weight bands for thorax/abdomen CT, while age bands are frequently used for head CT [9]. In the present retrospective multicentre dataset, patient weight (and other size surrogates) was not consistently recorded across all participating hospitals; therefore, age-based grouping was adopted to ensure consistent stratification across centres and sufficient numbers per category. The four age categories (< 1, 1–4, 5–10 and 11–18 years) approximate commonly used international paediatric groupings (< 1, 1– < 5, 5– < 10, 10– < 15 years) while reflecting local paediatric service coverage up to 18 years [9].

For each hospital, CT protocol, and age group, the hospital's typical value was defined as the median CTDI_vol_ and DLP across eligible patients. Proposed national DRLs were then defined as the 75th percentile of these hospital-specific values across participating centres for each protocol and age group.

Inclusion and exclusion criteria: Examinations were included if (i) patient age at scanning was ≤ 18 years; (ii) the CT was performed between December 2023 and September 2025; (iii) the RIS/PACS examination label matched one of the six predefined protocols (brain, chest, abdomen–pelvis, chest–abdomen–pelvis, sinuses, and contrast-enhanced neck soft tissue); and (iv) CTDI_vol_ and DLP were available. Records were excluded if dose indices were missing or the examination did not match the predefined protocol categories. Where multiple eligible CT examinations were identified for the same patient within the study period, only the first eligible examination was retained to avoid repeated measures. Patient weight, clinical indication, and sedation status were not consistently available across centres and were therefore not used for stratification.

Prior to analysis, extracted dose indices and acquisition parameters were screened for completeness and plausibility using standardised variable definitions across sites. Outlying or implausible values were flagged and verified against the original PACS record and/or scanner dose report to confirm protocol classification and dose indices, and records with incomplete dose data were excluded.

All statistical analyses were performed using IBM SPSS Statistics (version 31; IBM Corp., Armonk, NY, USA), and figures were generated using GraphPad Prism (version 10; GraphPad Software, San Diego, CA, USA). As dose indices were not assumed to follow a normal distribution, non-parametric hypothesis testing was applied. Inter-group differences in CTDIvol and DLP were assessed using the Kruskal–Wallis H test (independent samples), with statistical significance defined as *p* < 0.05 (two-sided). For each CT protocol (and analysed separately for CTDI_vol_ and DLP), two sets of Kruskal–Wallis tests were undertaken: (i) inter-hospital variation within each age group (Hospitals A–F compared for each age category); and (ii) inter-age-group variation within each hospital (four age categories compared within each hospital). As the Kruskal–Wallis test indicates whether at least one group differs, results were interpreted primarily from group medians and interquartile ranges; pairwise post-hoc testing was not performed. To investigate technical contributors to dose variation, univariable linear regression analyses were performed in SPSS with CTDI_vol_ and DLP as dependent variables and kVp, mAs, pitch, and slice thickness as individual predictors. Model performance was summarised using the coefficient of determination (R^2^), and statistical significance was assessed at *p* < 0.05.

To contextualise the proposed Jordanian DRLs, the derived 75th percentile CTDI_vol_ and DLP values were compared descriptively with published paediatric CT DRLs from Switzerland, Germany, South Korea, and the United Kingdom, acknowledging differences in methodology, scanner technology, and year of data collection.

#### Dose metrics

Two standard dosimetric indicators were considered:

- CTDI_vol_ (mGy): This metric reflects the average radiation output delivered within the scanned volume and is commonly used to characterise scanner performance during helical CT. CTDI_vol_ is derived by dividing the weighted CT dose index (CTDI_w_) by the pitch, as shown in Eq. (1) (AAPM Report No. 96 [10]):
  1 $$CTD{I}_{vol}= \frac{CTD{I}_{w}}{pitch}$$
  where CTDI_w_ denotes the weighted CT dose index, and pitch is defined as the table travel per gantry rotation divided by the total collimated beam width [10].
- DLP (mGy·cm): This index accounts for both radiation intensity and the length of the body region scanned, thereby representing the overall radiation exposure of a CT examination. DLP is calculated by multiplying CTDI_vol_ by the scan length (Eq. (2)) (AAPM Report No. 96 [10]):
  2 $$DLP=CTD{I}_{vol} x scan length$$

This parameter reflects the overall radiation burden to the patient by accounting for both the scanner output (CTDI_vol_) and the length of the body region covered [10].

## Results

### Patient and examination characteristics

A total of 3794 paediatric patients were included in this study, of whom 56.7% were male (n = 2152) and 43.3% were female (n = 1642). The sample was stratified into four age groups: < 1 year (29.5%), 1–4 years (21.5%), 5–10 years (23.1%), and 11–18 years (25.9%). The distribution of patients across the six participating hospitals was generally balanced, ranging from 12.0% to 23.9% per centre. Details of the patient demographics and hospital contributions are summarised in Table 1.

**Table 1:** Distribution of patients by hospital, sex, and age group

Variable	Category	Frequency (%)
Hospital	A	640 (16.9%)
	B	636 (16.8%)
	C	643 (16.9%)
	D	516 (13.6%)
	E	905 (23.9%)
	F	454 (12.0%)
Sex	Male	2152 (56.7%)
	Female	1642 (43.3%)
Age Group	< 1 year	1118 (29.5%)
	1–4 years	817 (21.5%)
	5–10 years	876 (23.1%)
	11–18 years	983 (25.9%)

The age distribution by hospital is shown in Table 2, while Table 3 summarises the number of CT examinations performed across anatomical regions and age groups. This distribution supports the representativeness of the dataset and ensures sufficient coverage to establish reliable DRLs.

**Table 2:** Distribution of paediatric patients by hospital and age group

Hospital	< 1 year	1–4 years	5–10 years	11–18 years	Total
A	160	160	160	160	640
B	182	126	157	171	636
C	161	167	154	161	643
D	232	93	94	97	516
E	279	164	197	265	905
F	104	107	114	129	454
Total	1118	817	876	983	3794

**Table 3:** Distribution of CT examinations by anatomical region and age group

Examination	< 1 year	1–4 years	5–10 years	11–18 years	Total
Brain	441	309	299	371	1420
Chest	179	104	96	103	482
Neck Soft Tissue (w/c)	123	116	112	111	462
Sinuses	117	114	124	117	472
Abdomen–Pelvis	136	88	122	140	486
Chest–Abdomen–Pelvis	122	86	123	141	472
Total	1118	817	876	983	3794

*(w/c): with contrast media

### Dose metrics and proposed DRLs

**Fig. 1:**
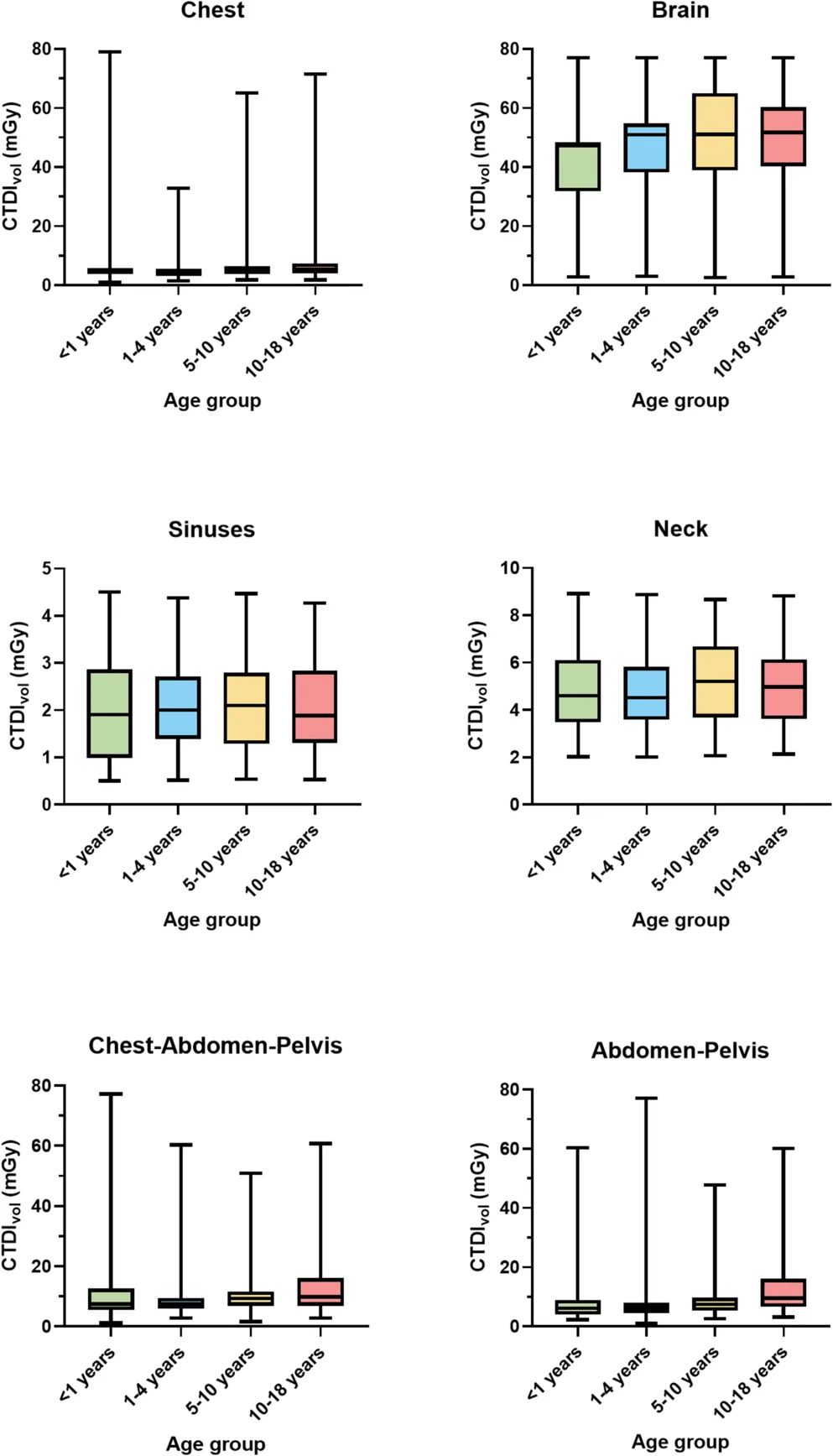
Box plots of CTDI_vol_ (mGy) for six paediatric CT examinations by age group, combining data from all hospitals. The plots display the median, interquartile range, and full range of CTDI_vol_ values for each protocol. Whiskers represent the minimum and maximum values; outlying values were reviewed against source PACS/RIS records prior to analysis

**Fig. 2:**
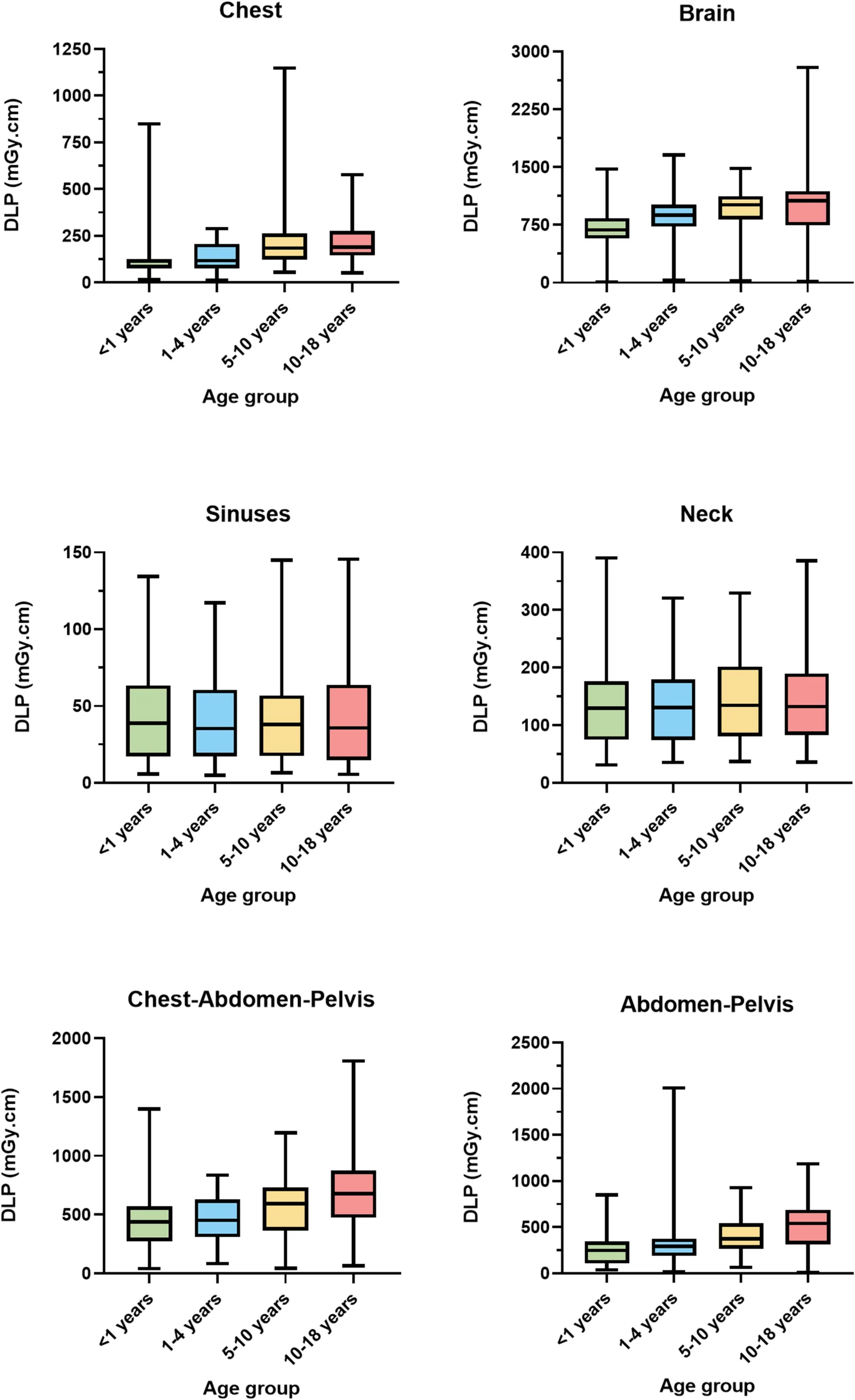
Box plots of DLP (mGy·cm) for six paediatric CT examinations by age group, combining data from all hospitals. The plots display the median, interquartile range, and full range of DLP values for each protocol. Whiskers represent the minimum and maximum values; outlying values were reviewed against source PACS/RIS records prior to analysis

The proposed DRLs for each CT examination were defined as the 75th percentile of the distribution of institutional (hospital-level) median CTDI_vol_ and DLP values across the six participating hospitals for each protocol and age group. Accordingly, Table 4 reports the 25th percentile, median, and 75th percentile of these institutional medians, with the 75th percentile representing the proposed national DRL. Pooled patient-level dose distributions are presented separately in Figs. 1 and 2 to illustrate within-group variability and outliers.

**Table 4:** Quartiles of institutional (hospital-level) median CTDI_vol_ (mGy) and DLP (mGy∙cm) values across six hospitals, by protocol and age group (75th percentile = proposed national DRL)

CT protocol	Age group	CTDI_vol_ (mGy)			DLP (mGy∙cm)		
		25th	Median	75th	25th	Median	75th
Brain	< 1 year	31.82	37.51	47.88	572.94	683.27	836.41
	1–4 years	38.16	50.93	54.79	726.05	875.06	1011.05
	5–10 years	38.82	51.09	65.02	820.60	1009.50	1123.07
	11–18 years	40.27	51.70	60.21	743.80	1061.80	1189.70
Chest	< 1 year	3.79	5.17	5.63	74.00	86.00	126.00
	1–4 years	3.00	3.90	5.63	76.10	117.55	207.45
	5–10 years	3.67	4.79	6.40	122.39	185.05	261.95
	11–18 years	3.95	5.30	7.34	144.40	198.90	275.90
Neck Soft Tissue (w/c)	< 1 year	3.48	4.60	6.11	74.80	129.40	176.30
	1–4 years	3.58	4.51	5.81	74.17	130.80	179.55
	5–10 years	3.68	5.21	6.69	80.90	134.60	201.90
	11–18 years	3.62	4.97	6.14	83.00	132.50	189.80
Sinuses	< 1 year	0.99	1.90	2.86	17.35	38.80	63.25
	1–4 years	1.38	2.00	2.70	17.35	35.35	60.55
	5–10 years	1.29	2.10	2.79	17.57	38.00	56.80
	11–18 years	1.30	1.88	2.83	14.85	35.80	63.80
Abdomen–Pelvis	< 1 year	4.06	6.11	8.88	105.07	246.35	345.10
	1–4 years	4.57	6.22	8.02	186.60	289.90	373.83
	5–10 years	5.36	7.46	9.70	264.85	370.95	540.92
	11–18 years	6.60	9.52	16.12	316.55	538.95	689.27
Chest–Abdomen–Pelvis	< 1 year	5.49	7.50	12.65	274.72	437.60	571.32
	1–4 years	6.04	7.48	9.52	306.98	451.40	630.92
	5–10 years	6.84	9.32	11.61	365.95	592.10	728.50
	11–18 years	6.89	9.80	16.12	474.90	677.20	875.45

Notable increases in radiation dose with patient age were observed for most examinations. For example, in abdomen–pelvis CT, the institutional median CTDI_vol_ increased from 6.11 mGy in infants (< 1 year) to 9.52 mGy in adolescents (11–18 years), while the corresponding institutional median DLP values rose from 246.35 to 538.95 mGy·cm. Similarly, for brain CT, the median of institutional median CTDI_vol_ increased from 37.51 mGy in infants (< 1 year) to 51.70 mGy in adolescents (11–18 years), with a corresponding increase in the median institutional DLP from 683.27 to 1061.80 mGy·cm.

In chest CT, the institutional median CTDI_vol_ increased moderately with age (from 5.17 to 5.30 mGy), whereas DLP showed a more notable increase (from 86.00 to 198.90 mGy cm). The chest–abdomen–pelvis protocol recorded the highest overall DLP values among all examinations, with a median DLP of 437.60 mGy·cm in infants and 677.20 mGy·cm in adolescents. Median CTDI_vol_ for chest–abdomen–pelvis increased from 7.50 to 9.80 mGy.

In contrast, dose values for neck soft tissue and sinuses CT remained relatively stable across age groups. The proposed DRLs (75th percentile values) for sinuses CT were consistent, ranging from 2.70 to 2.86 mGy for CTDI_vol_ and from 56.80 to 63.80 mGy·cm for DLP.

These findings support the establishment of national DRLs for six commonly performed paediatric CT protocols in Jordan and highlight the importance of age-specific dose optimisation, particularly in higher-dose examinations such as brain, abdomen–pelvis, and chest–abdomen–pelvis.

### Dose variation

Inter-hospital (Hospitals A–F) and within-hospital (between age categories within the same hospital) comparisons, assessed using Kruskal–Wallis tests, demonstrated statistically significant variability in CTDI_vol_ and DLP for several protocols. For clarity, inter-hospital p-values refer to comparisons across Hospitals A–F performed separately within each age group, whereas within-hospital p-values refer to comparisons across age groups performed separately within each hospital.

Overall, the largest between-hospital differences were observed for brain, chest, abdomen–pelvis, and chest–abdomen–pelvis examinations.

For brain CT, inter-hospital differences in CTDIvol and DLP were statistically significant in every age group (all *p* < 0.001). Among infants (< 1 year), the institutional median CTDIvol ranged from 28.24 mGy (Hospital D) to 58.80 mGy (Hospital F), and the institutional median DLP ranged from 399.7 mGy·cm (Hospital D) to 1030.85 mGy·cm (Hospital A). Within-hospital age-group comparisons indicated significant differences across age categories at Hospitals B, D, E and F (*p* < 0.001), whereas age-related differences were not statistically significant at Hospitals A and C (CTDI_vol_: *p* = 0.298 and *p* = 0.632; DLP: *p* = 0.544 and *p* = 0.333). This corresponds to an approximately twofold difference between hospitals in infant brain CTDI_vol_ and an approximately 2.6-fold difference in infant brain DLP, indicating clinically meaningful variation in typical practice.

In abdomen–pelvis CT, inter-hospital variation was statistically significant for both CTDIvol and DLP in every age group (all *p* < 0.001). For adolescents (11–18 years), institutional median CTDI_vol_ values ranged from 5.80 mGy (Hospital F) to 16.13 mGy (Hospital B), while institutional median DLP values ranged from 247.3 mGy·cm (Hospital F) to 767.0 mGy·cm (Hospital B). Within-hospital age-group comparisons were significant at Hospitals A, B, D, E and F (*p* ≤ 0.002), but not at Hospital C (CTDI_vol_: *p* = 0.733; DLP: *p* = 0.164), indicating inconsistent age adaptation between centres.

For chest CT, inter-hospital variation was statistically significant across age groups for CTDIvol (*p* = 0.006) and for DLP (all *p* < 0.001). Hospital F reported the highest institutional medians across multiple age categories (e.g., infants: CTDI_vol_ 30.48 mGy; DLP 553.10 mGy·cm). Within-hospital age-group comparisons were statistically significant for CTDI_vol_ at all hospitals (*p* ≤ 0.025). For DLP, age-group differences were statistically significant at Hospitals A, B, C, E and F (*p* ≤ 0.007) but not at Hospital D (*p* = 0.380).

Chest–abdomen–pelvis CT exhibited substantial between-hospital differences, with statistically significant inter-hospital variation for both CTDI_vol_ and DLP in every age group (all *p* < 0.001). In infants (< 1 year), institutional median CTDIvol ranged from 3.06 mGy (Hospital E) to 60.33 mGy (Hospital F), while institutional median DLP ranged from 69.68 mGy·cm (Hospital E) to 587.6 mGy·cm (Hospital C). Within-hospital age-group comparisons were statistically significant for CTDI_vol_ in four hospitals (A, B, D and F; *p* ≤ 0.014) and for DLP in five hospitals (A, B, D, E and F; *p* ≤ 0.044), with Hospitals C and E showing no statistically significant age-group differences in CTDI_vol_, and Hospital C also showing no statistically significant age-group differences in DLP. The infant chest–abdomen–pelvis protocol showed the greatest between-centre spread, with an approximately 20-fold difference in institutional median CTDI_vol_ across hospitals, reinforcing the need to standardise protocol in smaller children.

For neck soft tissue CT (with contrast), inter-hospital differences were not statistically significant across age groups for CTDI_vol_ or DLP (*p* = 0.192 and p = 0.694), and within-hospital age-group comparisons were also not statistically significant (all *p* > 0.05). This suggests comparatively consistent protocol implementation for this examination across participating centres.

For sinuses CT, inter-hospital variation was not statistically significant for CTDIvol or DLP (*p* = 0.632 and *p* = 0.901, respectively), and within-hospital age-group comparisons were also not statistically significant (all *p* > 0.05). Dose values for this protocol were comparatively low relative to body CT examinations.

Collectively, these findings indicate marked between-centre variability for several high-dose protocols, reinforcing the need for protocol harmonisation and ongoing dose optimisation.

To further illustrate the variation in dose by age group in the pooled national dataset, Figs. 1 and 2 present box plots of CTDIvol and DLP values, respectively, across the four paediatric age categories for each CT protocol. These plots demonstrate age-related increases in DLP for body protocols (e.g., chest, chest–abdomen–pelvis, and abdomen–pelvis), with greater variability observed in body examinations. As DLP incorporates both CTDI_vol_ and scan length (Eq. 2), age-related increases in DLP may reflect changes in scan range and/or scanner output.

### Acquisition parameters and predictors

Substantial variation was observed across hospitals, reflecting differences in scanner models, protocol design, and radiologist preferences. For example, the kVp ranged from 76 to 140 kVp across sites, while mAs values spanned a wide range (e.g., 10–392 mAs for brain CT at Hospital D). Similarly, pitch values varied from 0.2 to 1.7, and slice thickness ranged from 1.0 to 7.0 mm, depending on the protocol and hospital.

Notably, even for the same examination, inter-institutional variability in scan settings was pronounced. For example, abdomen–pelvis CT protocols ranged from 1.0 to 7.0 mm in slice thickness, 42–583 mAs, and pitch values from 0.2 to 1.2, as presented in Table 5.

**Table 5:** Ranges of acquisition parameters across CT protocols and hospitals

CT examination	kVp	mAs	Pitch factor	FOV (mm)	Slice thickness
Hospital A					
Brain	100–120	201–397	0.901–1.200	120–199	2.5–3.0
Chest	80–120	61–198	0.901–1.19	161–239	2.0–2.5
Neck Soft Tissue (w/c)	80–120	106–344	0.291–0.902	140–199	1–1.5
Sinuses	80–120	20–99	0.903–1.19	120–198	2.0–3.0
Abdomen–Pelvis	80–120	101–348	0.295–0.904	160–247	2.5–3.0
Chest–Abdomen–Pelvis	80–120	249–399	0.904–1.198	183–278	2.0–3.0
Hospital B					
Brain	100–140	100–448	0.2–1.01	123–586	1.0–5.0
Chest	80–120	26–399	0.55–1.20	115–450	1.0–5.0
Neck Soft Tissue (w/c)	80–120	102–275	0.902–1.094	121–184	1.0–5.0
Sinuses	80–120	21–111	1.01–1.452	126–188	2.0–3.0
Abdomen–Pelvis	76–120	103–339	0.2–1.2	123–586	1.0–5.0
Chest–Abdomen–Pelvis	100–120	110–400	0.55–1.2	122–770	1.0–5.0
Hospital C					
Brain	100–120	182–379	0.907–1.199	130–189	2.5–3.0
Chest	80–120	70–179	0.911–1.19	190–277	2.0–3.0
Neck Soft Tissue (w/c)	80–120	90–318	0.901–1.20	140–199	1.0–1.50
Sinuses	80–120	25–89	0.902–1.19	130–189	2.0–3.0
Abdomen–Pelvis	80–120	122–319	0.908–1.198	170–257	2.50–3.0
Chest–Abdomen–Pelvis	100–120	160–347	0.905–1.196	190–277	2.0–3.0
Hospital D					
Brain	80–120	10–392	0.50–1.70	230–333	1.0–7.0
Chest	100–120	10–165	0.55–1.20	80–420	1.0–5.0
Neck Soft Tissue (w/c)	80–100	82–159	0.903–1.09	141–199	1.0–1.50
Sinuses	100–120	10–165	0.55–1.20	80–420	1.0–5.0
Abdomen–Pelvis	80–140	42–281	0.55–1.20	98–546	1.0–7.0
Chest–Abdomen–Pelvis	80–120	17–361	0.55–1.20	80–675	1.0–5.0
Hospital E					
Brain	100–120	182–379	0.907–1.19	130–189	2.5–3.0
Chest	80–120	70–179	0.91–1.199	160–227	2.0–2.5
Neck Soft Tissue (w/c)	80–120	90–318	0.901–1.20	140–199	1.0–1.50
Sinuses	80–120	130–189	0.902–1.191	130–189	2.0–3.0
Abdomen–Pelvis	80–120	122–319	0.908–1.198	170–257	2.5–3.0
Chest–Abdomen–Pelvis	100–120	160–347	0.905–1.196	190–277	3.0–3.0
Hospital F					
Brain	100–120	37–280	0.5–1.5	105–451	1.0–5.0
Chest	100–120	110–365	0.5–1.00	200–723	1.0–5.0
Neck Soft Tissue (w/c)	100–120	200–349	0.901–1.09	140–199	1.0–1.5
Sinuses	100–120	70–119	1.20–1.5	140–199	2.0–3.0
Abdomen–Pelvis	100–120	30–320	0.5–1.00	192–580	2.0–5.0
Chest–Abdomen–Pelvis	100–120	30–320	0.5–1.00	192–580	2.0–5.0

All four acquisition parameters were significantly associated with dose metrics (*p* ≤ 0.001). The R^2^ from univariable regression analyses are presented in Table 6.

**Table 6:** Univariable linear regression (R^2^) between acquisition parameters (kVp, mAs, pitch, slice thickness) and CTDI_vol_/DLP

	Predictor	R^2^	*P*-value
CTDI_vol_	mAs	0.473	< 0.001
	Pitch	0.561	< 0.001
	Slice thickness	0.597	< 0.001
	kVp	0.603	< 0.001
DLP	mAs	0.562	< 0.001
	Pitch	0.616	< 0.001
	Slice thickness	0.626	0.001
	kVp	0.630	0.001

Among the predictors, kVp demonstrated the strongest relationship with both CTDI_vol_ (R^2^ = 0.603) and DLP (R^2^ = 0.630), followed closely by slice thickness and pitch. While mAs had a weaker association, it remained a statistically significant predictor.

These findings indicate that variability in acquisition protocols was associated with variation in patient dose indices (or contributed to variation and support the need for national standardisation of acquisition parameters. Optimising kVp selection, limiting excessive slice thickness, and avoiding unnecessarily low pitch values may help reduce dose without compromising diagnostic quality.

### International comparison of DRLs

As presented in Tables 7 and 8, dose values showed considerable inter-country variability, particularly for high-dose protocols such as brain and abdomen–pelvis CT. For example, the CTDI_vol_ for brain CT in the 5–10 years group was 65.02 mGy in Jordan, compared to 40 mGy in Switzerland and 44.1 mGy in South Korea. Similarly, the abdomen–pelvis DLP for the 11–18 years group was 689.27 mGy·cm in Jordan, compared with 780 mGy·cm reported for South Korea; however, UK national paediatric NDRLs do not provide paediatric abdomen–pelvis CT values, and the German paediatric DRWs report CTDIvol with indicative scan lengths (i.e., paediatric DLP is not explicitly listed) [6, 11, 12].

**Table 7:** International comparison of 75th percentile CTDI_vol_ (mGy) values for paediatric CT examinations across countries and age groups. The bracketed numbers (e.g., [8, 9]) indicate the reference sources from which the DRL values for each country were obtained

CT examination	Age group	Switzerland [13]	Germany [12]	South Korea [14]	United Kingdom [6, 11]	Jordan (current study)
Brain	< 1 year	20	25	39.1	19	47.88
	1–4 years	30	34	41.7	24	54.79
	5–10 years	40	40	44.1	36	65.02
	11–18 years	–	45*	55.3	46 − 47*	60.21
Chest	< 1 year	5	1.0 − 1.5*	5.7	Weight-banded	5.63
	1–4 years	8	2.0	6.8	Weight-banded	5.63
	5–10 years	10	3.0	9.3	Weight-banded	6.40
	11–18 years	–	5.0*	13.8	Weight-banded	7.34
Neck Soft Tissue (w/c)	< 1 year	–	–	–	–	6.11
	1–4 years	–	–	–	–	5.81
	5–10 years	–	–	–	–	6.69
	11–18 years	–	–	–	–	6.14
Sinuses	< 1 year	–	–	–	–	2.86
	1–4 years	–	–	–	–	2.70
	5–10 years	–	–	–	–	2.79
	11–18 years	–	–	–	–	2.83
Abdomen–Pelvis	< 1 year	–	–	7.5	–	8.88
	1–4 years	–	–	8.9	–	8.02
	5–10 years	–	4.0	12.4	–	9.70
	11–18 years	–	7.0	16.6	–	16.12
Chest–Abdomen–Pelvis	< 1 year	–	–	–	–	12.65
	1–4 years	–	–	–	–	9.52
	5–10 years	–	–	–	–	11.61
	11–18 years	–	–	–	–	16.12

*UK values are from UKHSA National Diagnostic Reference Levels (NDRLs) (adopted 2022). Paediatric head NDRLs are age-banded (0– < 1, 1– < 5, 5– < 10, 10– < 15 years); for 15– < 18 years, adult head CT values are applied. Paediatric chest NDRLs are weight-banded (0– < 5 kg, 5– < 15 kg, 15– < 30 kg, 30– < 50 kg, 50– < 80 kg); therefore, direct comparison to age-banded results is limited. Germany's values are from the Bundesamt für Strahlenschutz (BfS) diagnostic reference values (DRW) (dated 17 Nov 2022; published 11 Jan 2023). Paediatric CT DRWs are reported as CTDI_vol_ with indicative scan length; DLP is not reported for paediatric CT in this publication. Age/weight categories extend to 10– < 15 years; older adolescents may be classified as adult DRWs

**Table 8:** International comparison of 75th percentile DLP (mGy·cm) values for paediatric CT examinations across countries and age groups

CT examination	Age group	Switzerland [13]	Germany [12]	South Korea [14]	United Kingdom [6, 11]	Jordan (current study)
Brain	< 1 year	270	–	545	290	836.41
	1–4 years	420	–	508	420	1011.05
	5–10 years	560	–	798	570	1123.07
	11–18 years	–	–	947	690 − 790*	1189.70
Chest	< 1 year	110	–	53	Weight-banded	126.00
	1–4 years	200	–	121	Weight-banded	207.45
	5–10 years	220	–	160	Weight-banded	261.95
	11–18 years	–	–	473	Weight-banded	275.90
Neck Soft Tissue (w/c)	< 1 year	–	–	–	–	176.30
	1–4 years	–	–	–	–	179.55
	5–10 years	–	–	–	–	201.90
	11–18 years	–	–	–	–	189.80
Sinuses	< 1 year	–	–	–	–	63.25
	1–4 years	–	–	–	–	60.55
	5–10 years	–	–	–	–	56.80
	11–18 years	–	–	–	–	63.80
Abdomen–Pelvis	< 1 year	–	–	196	–	345.10
	1–4 years	–	–	338	–	373.83
	5–10 years	–	–	513	–	540.92
	11–18 years	–	–	780	–	689.27
Chest–Abdomen–Pelvis	< 1 year	–	–	–	–	571.32
	1–4 years	–	–	–	–	630.92
	5–10 years	–	–	–	–	728.50
	11–18 years	–	–	–	–	875.45

*See the footnote to Table 7 for details regarding UK and German DRL categorisation and comparison limitations (age- versus weight-banding, and paediatric DLP reporting)

These findings underscore the dose variability across nations, likely influenced by differences in scanner models, imaging protocols, and national dose optimisation policies. While Jordan’s proposed DRLs for several protocols fall within the international range, higher values were observed for brain CT compared with Switzerland and the UK; chest CT comparisons with the UK should be interpreted cautiously because UK paediatric chest NDRLs are weight‑banded rather than age‑banded.

## Discussion

This multicentre study proposed updated national DRLs for six paediatric CT examinations in Jordan, encompassing brain, chest, abdomen–pelvis, chest–abdomen–pelvis, sinuses, and neck soft tissue protocols. By including 3794 patients across six hospitals, this work provides one of the largest multicentre datasets reported in Jordan and provides age-specific DRLs that reflect typical practice across the country. The results demonstrated clear dose escalation with patient age in body examinations, while brain and sinus CT protocols showed more stable trends. Importantly, the proposed DRLs highlight both areas of alignment with international standards and protocols where further optimisation is required, particularly in brain and chest imaging [5]. A recently published national multicentre survey focusing specifically on paediatric head CT in Jordan also reported substantial inter-hospital variability in CTDI_vol_ and DLP and highlighted the need for national dose standardisation; our findings for brain CT are consistent with this overall national concern, while the present work extends the scope by establishing DRLs across six routine paediatric CT protocols and evaluating technical predictors of dose variation [8].

Consistent with this, inter-hospital dose variability was pronounced, especially for high-dose examinations such as brain, abdomen–pelvis, and chest–abdomen–pelvis CT. In some instances, median CTDI_vol_ values differed more than threefold between hospitals, underscoring the lack of harmonisation in protocol design and scanner operation. Although dose increases with age were expected due to larger body sizes and longer scan ranges, the degree of variation between hospitals suggested inconsistent adjustment of exposure parameters. This finding emphasises the need for stronger national guidance and quality assurance frameworks to reduce disparities and ensure equitable radiation protection for paediatric patients [15].

Analysis of acquisition parameters confirmed that variability in scan settings contributed significantly to dose differences. Substantial variation in kVp, mAs, pitch, and slice thickness was observed, with regression analysis identifying kVp, slice thickness, and pitch as the strongest predictors of CTDI_vol_ and DLP. Hospitals employing low pitch values and larger slice thicknesses often recorded higher dose levels. These results reinforce the importance of tailored paediatric protocols and the consistent use of dose-saving technologies (e.g., AEC and iterative reconstruction) are recognised optimisation tools; however, their utilisation could not be assessed in this dataset [16].

Comparison with international data revealed that Jordanian DRLs for abdomen–pelvis and chest–abdomen–pelvis CT were generally comparable with those reported in Switzerland, Germany, South Korea, and the United Kingdom [13, 14, 17, 18]. However, DRLs for brain CT were consistently higher than international benchmarks, exceeding values from Switzerland and South Korea by 10–20 mGy in some age groups. Similarly, chest CT doses, particularly in younger children, were elevated compared with international values. These discrepancies are likely attributable to differences in scan length, operator practices, and incomplete adaptation of protocols for smaller patients [19]. The findings highlight the need for closer alignment with international optimisation strategies and reinforce the ICRP recommendation for regular review of DRLs every three to five years [5].

Overall, the establishment of national DRLs provides a critical tool for improving radiation protection in paediatric CT. These benchmarks allow institutions to assess their performance against national norms, identify protocols where dose levels are excessive, and initiate local reviews [20]. In particular, elevated brain and chest CT values warrant targeted interventions, while the observed inter-hospital variability suggests that harmonisation of acquisition protocols should be prioritised. Training radiologists and technologists in paediatric-specific dose optimisation, together with routine audits and quality assurance, will be essential for sustaining improvements [21].

The strengths of this study include its large sample size, multicentre design, and inclusion of six different CT protocols across four age groups. The integration of regression analysis provided new insights into the technical determinants of dose variation, supporting practical recommendations for optimisation. Nonetheless, certain limitations should be noted. Effective dose and image quality were not assessed, which limited direct evaluation of clinical risk–benefit balances. Patient weight, clinical indication, and sedation status were not consistently available from PACS/RIS across all centres; therefore, analyses were age-based and did not control for these factors. Most contrast-enhanced examinations were not included because of insufficient patient numbers, which reduces the generalisability of findings to such protocols. Differences in scanner technology and availability of dose reduction features across hospitals may also have influenced results.

The proposed national paediatric DRLs provide benchmark values that can be implemented in routine quality assurance programmes. Centres with typical doses exceeding the proposed DRLs should undertake local protocol review (e.g., scan range, kVp selection, pitch, and reconstruction settings) to identify remediable contributors to elevated CTDI_vol_ and/or DLP. Periodic re-audit is recommended to monitor the impact of optimisation and to support national harmonisation of paediatric CT practice.

International comparisons were limited by variations in methodology, sample size, and year of data collection, which complicates direct equivalence of DRLs across countries [22]. Image quality and diagnostic adequacy were not assessed; therefore, dose comparisons are not directly linked to clinical performance. Further limitation is that patient stratification was age-based rather than weight- or size-based. For trunk CT examinations, patient weight (or other size surrogates) may better reflect attenuation and protocol selection, and age bands can increase within-group variability and limit comparability with DRL systems that use weight bands. Future national DRL updates should incorporate routinely recorded patient weight and/or size surrogates (e.g., SSDE) to enable weight- and/or size-stratified DRLs for body CT protocols [9].

## Conclusion and future directions

This multicentre study established national DRLs for paediatric CT in Jordan, providing benchmarks that reflect current clinical practice and identify areas for further optimisation. While many values were comparable with international standards, elevated doses in brain and chest CT examinations, along with notable inter-hospital variability, underscore the need for protocol harmonisation and stronger adherence to paediatric-specific optimisation strategies. These findings offer an evidence base to support national quality assurance programmes and ongoing initiatives aimed at improving radiation protection in paediatric imaging.

Future research should incorporate effective dose estimation and image quality assessment to balance dose optimisation with diagnostic adequacy. Expanding DRL surveys to include smaller regional and private hospitals would enhance the representativeness of national benchmarks. The development of a centralised dose registry, supported by automated dose-tracking systems, would facilitate continuous data collection and periodic DRL review in accordance with ICRP recommendations. In parallel, greater emphasis on education and training for radiologists and technologists, together with the adoption of advanced dose-reduction technologies, will be essential for achieving sustained improvements in paediatric CT practice nationwide.

## Data Availability

The datasets used and/or analysed during the current study are available from the corresponding author on reasonable request.
